# Prebiotics and synbiotics – *in ovo* delivery for improved lifespan condition in chicken

**DOI:** 10.1186/s12917-018-1738-z

**Published:** 2018-12-17

**Authors:** M. Siwek, A. Slawinska, K. Stadnicka, J. Bogucka, A. Dunislawska, M. Bednarczyk

**Affiliations:** 1 Department of Animal Biotechnology and Genetics, UTP University of Science and Technology, Mazowiecka, 28 85-084 Bydgoszcz, Poland; 2 Department of Animal Physiology, Physiotherapy and Nutrition, UTP University of Science and Technology, Mazowiecka, 28 85-084 Bydgoszcz, Poland

**Keywords:** Broilers, Synbiotics, Hatching window, Embryonic development, Day 12 of egg incubation, Microflora, Performance, Intestinal morphology, Gene expression

## Abstract

Commercially produced chickens have become key food-producing animals in the global food system. The scale of production in industrial settings has changed management systems to a point now very far from traditional methods. During the perinatal period, newly hatched chicks undergo processing, vaccination and transportation, which introduces a gap in access to feed and water. This gap, referred to as the hatching window, dampens the potential for microflora inoculation and as such, prevents proper microbiome, gastrointestinal system and innate immunity development. As a consequence, the industrial production of chickens with a poor microbial profile leads to enteric microbial infestation and infectious disease outbreaks, which became even more prevalent after the withdrawal of antibiotic growth promoters on many world markets (e.g., the EU).

This review presents the rationale, methodology and life-long effects of *in ovo* stimulation of chicken microflora. *In ovo* stimulation provides efficient embryonic microbiome colonization with commensal microflora during the perinatal period. A carefully selected bioactive formulation (prebiotics, probiotics alone or combined into synbiotics) is delivered into the air cell of the egg on day 12 of egg incubation. The prebiotic penetrates the outer and inner egg membranes and stimulates development on the innate microflora in the embryonic guts. Probiotics are available after the mechanical breakage of the shell membranes by the chick’s beak at the beginning of hatching (day 19). The intestinal microflora after *in ovo* stimulation is potent enough for competitive exclusion and programs the lifespan condition. We present the effects of different combinations of prebiotic and probiotic delivered *in ovo* on day 12 of egg incubation on microflora, growth traits, feed efficiency, intestinal morphology, meat microstructure and quality, immune system development, physiological characteristics and the transcriptome of the broiler chickens.

We discuss the differences between *in ovo* stimulation (day 12 of egg incubation) and *in ovo* feeding (days 17–18 of egg incubation) and speculate about possible future developments in this field. In summary, decades of research on *in ovo* stimulation and the lifelong effects support this method as efficient programming of lifespan conditions in commercially raised chickens.

## Introduction

Commercially produced chickens, together with other poultry species, have become key food-producing animals within the global food system. Both egg and meat-type chickens provide easily achievable and affordable animal protein, which is particularly important for food security in developing countries. An increase in global food production is critical to meet the demand of the rapidly growing human population. The recent prognosis for overall growth in agriculture is expected to be between 25 and 70% by 2050 compared to current production rates [1]. Poultry production is predicted to double worldwide, especially in developing countries, which will require further intensification of the chicken food chain [2]. These increased production rates will be accompanied by serious quality adjustments, mainly in animal welfare and food safety. For this reason, anti-antibiotic strategies will continue, and the introduction of prebiotic and/or probiotic preparations will be in demand as safe and efficient methods to improve food production in a manner called “sustainable intensification.” Meeting all these challenges in the future will be possible with the further development of technologies of precision livestock farming for egg [3] and meat [4] poultry production.

One of the precision livestock farming tools in poultry production is *in ovo* technology for modulating the conditions inside the egg through nutrients, vaccines and other bioactives. It allows depositing a certain amount of a carefully selected substance into a specific site within an incubating egg. *In ovo* technology is focused on the most critical time in the bird’s development, which is the perinatal period. The perinatal period lasts from the final days of the egg’s incubation to the first days post-hatching. During that time, the embryo has to adjust to a change in diet (from fat-rich to carbohydrate-rich) and exposure to environmental microbes. In commercial settings, newly hatched chicks are first processed at the hatchery and then transported to the farm before having received any feed or water. Given the large numbers of industrially hatched chicks per batch, these procedures can take a significant amount of time (the so-called hatching window, discussed further in Chapter 4). The disadvantages of the hatching window are practically unavoidable given the constantly growing scale of poultry production. For this reason, *in ovo* technology has been developed to facilitate manipulation of the chicken embryo before hatching. In principal, it is based on the mechanical delivery of substances directly into the incubating egg. This technology was primarily established for the vaccination of 18-day-old embryos against multiple infectious agents, including the Marek’s disease virus and infectious bursal disease [5, 6]. Apart from vaccination, *in ovo* technology has been applied to the delivery of prebiotics, probiotics, synbiotics, vitamins, hormones, carbohydrates and peptides [7, 8]. Such precise manipulation of the embryo improves the robustness and resilience of the hatched chicks, which contributes to their further post-hatching development.

The literature describes two major time points of chicken embryo development that have been successfully used for substance delivery through *in ovo* technology. The first time point is around day 12 of egg incubation and has been used solely for the delivery of prebiotics and synbiotics. The deposition site at this stage of embryonic development is an air cell lined by two layers of egg membranes, which are in contact with the highly vascularized chorioallantoic membrane. The route mechanisms of prebiotic and synbiotic penetration through egg membranes are discussed in detail in Chapter 4. In principle, delivering prebiotics at this time point is aimed to stimulate native egg microflora. Pedroso et al. [9] has already proven that neonatal chicks possess microbiota within their intestinal tract even before hatching [9]. However, their diversity and abundance are not effective enough for the competitive exclusion of undesirable microorganisms. Nevertheless, these microbiota are most likely stimulated by *in ovo*-delivered prebiotic. Two independent studies have demonstrated that prebiotics administered *in ovo* on day 17 [10] and on days 12 or 17 [11] increased the number of bifidobacteria in newly hatched chicks. The second study especially clearly shows that the most effective time point for prebiotic delivery, defined by the number of bifidobacteria, is day 12 of egg incubation. When the bioactive formulation for *in ovo* delivery on day 12 of egg incubation is a synbiotic, then prebiotic stimulates native microflora from day 12 to 18 of egg incubation and probiotic is ingested on day 18 after the chick has started pipping. For this reason, the delivery of prebiotics and synbiotics on day 12 of egg incubation in chickens is called *in ovo* stimulation (as opposed to *in ovo* feeding, discussed further). This method has been developed and patented by K. Gulewicz and M. Bednarczyk [12].

The second time point for the *in ovo* delivery of bioactives is around days 17/18 of egg incubation. Then, the chicken embryo is completely developed and, until hatching, it will only grow, using the yolk as a source of nutrients. The perinatal period is crucial for the chick because it prepares it for life post-hatching. *In ovo* technology used at this time point aims to mitigate the negative effects of starvation during the hatching window. For this reason, the late-term chicken embryo is supplemented with nutrients sufficient for securing the newly hatched chick through the hatching window. This method is referred to as *in ovo* feeding and has been patented by Uni and Ferket [13]. The differences between *in ovo* stimulation and *in ovo* feeding are presented in Chapter 4. There have been trials to extend *in ovo* feeding to microbiome stimulation and competitive exclusion by injecting probiotics on day 17/18 of egg incubation, reviewed by Roto et al. [8]. The question could be asked here, what difference it makes for intestinal microflora development as long as the inoculation is done before hatching? In fact, the effect of the time point is tremendous when it comes to *in ovo* delivery of prebiotics and synbiotics. First, *in ovo* stimulation on day 12 of egg incubation is primarily based on the enhancement of growth of native flora present in the embryo by using prebiotics or synbiotics. Second, the injection site differs between day 12 and day 18 of egg incubation in embryos due to the size and structural changes of the egg. On day 12 of egg incubation, the site of *in ovo* injection is the air cell, which is safe for the embryo and easy to automate. A late-term embryo is more likely to be traumatized by an improperly performed injection. The injection site can be the amnion or the embryo, depending on the chicken embryo position in the egg. Third, *in ovo* feeding on day 18 of egg incubation is likely to reduce hatchability, unlike *in ovo* stimulation on day 12 of egg incubation, which leaves hatchability rates practically intact [7, 8].

In light of these considerations, this review aims to present the paradigms of *in ovo* stimulation of the embryonic microbiome in chicks, performed on day 12 of egg incubation, and its long-term effects. We discuss the development of the chicken microbiome (Chapter 2), means of its modulation (Chapter 3), various issues regarding *in ovo* microbiome stimulation (Chapter 4) and how it influences lifespan condition (Chapter 5). We also outline what lies ahead in microbiome research in poultry and what possible steps can be taken to forward the research into and application of *in ovo* microbiome stimulation (Chapter 6).

### Development of the chicken microbiome

The microbiome is defined as the total of the genetic information provided by a community of microorganisms (microbiota) inhabiting one environment, for example, intestinal mucosa and lumen in animals. Microbiota consist of commensal, symbiotic and pathogenic microorganisms. Recently, the most accurate analysis of the chicken gastrointestinal tract (GIT) microbiome was done by Wei et al. [14]. According to these authors, there are 915 species defined as operational taxonomic units (OTUs) in the chicken GIT. Among them, there is a high prevalence of Firmicutes (70%), represented by 495 OTU, Bacteroidetes (12.3%), represented by 139 OTU and Proteobacteria (9.3%), represented by 124 OTU. Most of the genera identified in the phyla *Firmicutes, Proteobacteria, Bacteroidetes* are common intestinal residents. However, some intriguing genera were also identified, e.g., *Ethanoligenens—an ethanol-producing bacteria (phylum Firmicutes).* The chicken microbiome depends on diet, location and age. Therefore, the taxonomic characteristic of each GIT section varies between the studies and also differs in the conditions of hatching and rearing [15]. The microbiome composition differs also between the morphological and physiological sections of the GIT, with its diversity and abundance increasing towards the hindgut. In birds, double ceca are the functional equivalent of the mammalian large intestine and an important site for fermentation. Detailed information on the most abundant taxa in individual sections of chicken GIT was reviewed by Stanley et al. [16]

An earlier opinion that the internal environment of the developing egg is sterile [17] was recently widely questioned, thanks to next-generation sequencing, which allows an investigation of whole microbial communities. For example, some bacteria can affect chicken embryos via infection of the female chicken’s reproductive organs, resulting in incorporation of bacteria into the egg during oogenesis [18]. Furthermore, the profile of gut microflora becomes more differentiated and the population number of particular groups becomes higher with the age of embryos [19].

In comparison to mammals, poultry has shorter GIT and faster digesta transit. This feature significantly influences the diversity of the birds’ microbiome compared to other livestock animals [20]. In the first day after hatching, the digestive system is the most intensively developing organ in birds. The microbiome of the newly hatched chick develops rapidly from days 1 to 3 post-hatching [21]. Such quick development is facilitated by the fast movement of the intestinal content, including bacteria. Therefore, the rapid development of the chicken microbiome is characterized by the quick multiplication of intestinal bacteria and their ability to adhere to the mucosa. In the ceca, the intestinal content slows, which promotes the development of microorganisms and the formation of the proper intestinal microbiome [22].

Colonization of the GIT with bacterial microflora is a key factor in the development and regulation of immunity, digestion, absorption of nutrients and their metabolism. The beneficial intestinal microflora helps not only in the digestion of food compounds but also reduces the potential of pathogen colonization in the guts. The entire process of microbiota development is stimulated by a huge number of different factors, including feed intake, antibiotics, supplements and other nutraceuticals, enzyme activities, age of the host, genetic modification and the whole environment [23]. Stimulation of commensal microbiota as early as possible (discussed in Chapter 4 of this review) is important to life-long programming and development in animals [24]. The interaction between the microbiome and its host leads to the proliferation of beneficial bacteria and reduces the development of enteric pathogens in the chicken gut [25, 26].

In poultry nutrition, gastrointestinal microorganisms and their influence on the host are of great significance. Intestinal microflora can ensure a complementary source of exogenous vitamins. Bacteria inhabiting GIT have the ability to synthesize vitamin K as well as the majority of water-soluble B vitamins [27]. Beneficial microorganisms promote the development of intestinal layers of mucosa and epithelia. They are also capable of distribution of polysaccharides and provide amino acids and short-chain fatty acids (SCFA), which are an important energy source and metabolic modulators [28].

Intestinal microbiota are in constant cross-talk with the host. This is possible due to recognition between receptors present on the lining of the GIT and gut-associated lymphoid tissue (GALT) and the microbial ligands, called microbe-associated molecular patterns (MAMPs) [29]. Such interaction affects the immunological status and physiology of the individual host [22]. The continuous cross-talk between the host and intestinal microbiome is long term and contributes to maintaining homeostasis in the guts. The major role of commensal gut microbiota is to trigger the maturation of both innate defense mechanisms and adaptive immune response [30]. The impact of the chicken microbiome on innate and adaptive immunity is described in the review by Clavijo & Florez [15]. The host immune system continuously adapts to the intestinal microbiome. Intestinal epithelia and GALT can tell commensal microflora from pathogenic strains and activate inflammatory mechanisms only in response to the latter. This subtle recognition between commensals and pathogens allows for the stable growth of beneficial flora with instant and local immune response to pathogenic flora [29]. In this manner, animals contribute from fermentation products of the microbiome, but also eradicate any environmental pathogen that enters the GIT.

### Microbiome modulation

In nature, animals receive the first inoculation of the microbiota from the dam and as such, the microbiome can be passed to another generation. During hatching and directly post-hatching, chicks are exposed to hen’s microbiota present on the egg shell or in the litter, which serves as the first source of microbial inoculation [9]. Current breeding and production systems involve automated hatcheries that eliminate the contact between chicks and their dams. During chicken embryo development in the egg, the main route of colonization with environmental bacteria is from the air, through the pores of the egg shell into the embryo [17]. After hatching, initial inoculation with the bacteria is obtained while handling during chickens’ delivery from the hatchery to the farm. To avoid random composition of chicken microbiota, a planned microbiome stimulation might be introduced through direct supplementation of chicken embryo with proper bioactive substances (i.e., prebiotics and probiotics separately or combined into synbiotics).

Prebiotics are non-fermentable polysaccharides, which similarly to probiotics (discussed below) need to fulfil specific criteria to be qualified for GIT microbiome modulation. These compounds need to be resistant to digestion in the upper gastrointestinal tract, selectively stimulate beneficial microorganisms, and improve colonic microbiota composition. The most popular naturally occurring prebiotics are: fructans (e.g., fructooligosacchardies, short-chain fructooligosaccharides, oligofructose, inulin), followed by mannooligosaccharides (*Saccharomyces cerevisae*), soy oligosaccharides, and galacto- or transgalactooligosaccharides [31].

Most of the microorganisms used as probiotics for animals belong to various bacteria species, such as: *Lactobacillus*, *Bifidobacterium*, *Bacillus* or *Enterococcus*. Certain yeast (e.g., *Saccharomycces bourlardii* or *Saccharomyces cerevisae*) and fungal (e.g., *Aspergillus oryzae* or *Candida pintolepsii*) species can also be used for the same purpose. The variety of potential probiotics is tremendous. A document prepared by FAO lists 16 species and 57 strains of different microorganisms, which are used as separate strains or multispecies in a single product in animal nutrition [32]. Microorganism used as a probiotic need to fulfill particular requirements. It should be non-pathogenic to the future host organism and resistant to low pH and have a high concentration of bile acids. On top of that, from the manufacturing perspective, the potential probiotic should be easy to transport, store, and apply. The routine process of probiotic selection is based on several analyses covering: the in vitro challenge of low pH, toleration of an acid environment and ability to adhere to the intestinal epithelium [32]. Finally, when selecting potential probiotic for animal nutrition, one should make sure that it grows on an inexpensive medium. This last feature is of less importance when probiotic is applied using *in ovo* technology, where only a small amount of the bioactive substances is given (details are discussed in Chapter 4). Scientific reports show that probiotics applied in a standard way (as DFM, in-feed or in-water) improve the growth rate of the animals, increase efficiency and stimulate intestinal histomorphology (reviewed by FAO, [32]). However, the results obtained during feed trials are inconsistent and need to be verified on a case-by-case basis.

### *In ovo* stimulation of embryonic chicken microbiome

#### Principles of *in ovo* stimulation

In poultry practice, birds are offered prebiotics and probiotics at the hatchery by spray application or in the form of DFM (in-feed or in-water) during the first days post-hatching, after they have arrived at the farm. In this case, the whole embryonic and perinatal period (21 days) is disregarded. If we consider the total lifespan of a regular broiler chicken (42 days), applying DFM post-hatching seems like late intervention. The main concept of *in ovo* technology is to apply substances long before the bird hatches, which helps to program lifelong phenotypes (e.g., immunity, gut microbiome, performance, adaptive) already during the embryonic phase. In this manner, the *in ovo* strategy is focused solely on stimulating the colonization of embryonic GIT with native microbiota that will facilitate establishing the optimal microbiome already during egg incubation. We demonstrated that by delivering the volume of 0.2 ml of dissolved bioactive stimulus exactly on day 12 of egg incubation, we can initiate a whole cascade of events on different phenotypic levels, from gene expression modulation to growth performance. The timing of *in ovo* stimulation of microbial growth in embryonic GIT is quite crucial. If the *in ovo* injection into the air cell is performed earlier, then the bioactive cannot pass through the underdeveloped allantochorion, which at this point is not vascularized enough. If *in ovo* injection is performed on late-term embryos (day 17/18 of egg incubation), then the air cell can no longer be used and the injection site is the amnion or more often, the embryo muscles (refer to the Introduction for more details on the time point of *in ovo* stimulation). The timing of *in ovo* stimulation of microbiota colonization in embryonic GIT on day 12 of egg incubation was established experimentally based on measurements of the proliferation rates of Bifidobacteria at hatching. High proliferation rates were shown after injecting different doses of oligosaccharide prebiotic on day 12 of egg incubation, compared to days 1, 8 and 17 of egg incubation [11]. The doses of the bioactives for *in ovo* injections are determined empirically, based on the criterion of egg hatchability and intestinal bacteria abundance in 1-day-old chicks. Proper *in ovo* stimulation should result in high bacteria abundance in chicken meconium without dampening the hatchability of the injected eggs [33].

#### Mechanisms of *in ovo* stimulation by prebiotics and probiotics

Before we performed *in ovo* stimulation of the embryonic microflora on day 12 of egg incubation, *in ovo* technology had been routinely used on day 18 of egg incubation for *in ovo* vaccination or (more recently) *in ovo* feeding. The poultry community was convinced to keep using late-term embryos for injecting probiotics as well. The use of prebiotics *in ovo* was scarce. Our experimental data indicated day 12 of egg incubation as the most efficient time point to provide stimulation of embryonic microflora with no harm to the incubating eggs [11]. There are two groups of natural growth promoters that can be used for *in ovo* stimulation, i.e., prebiotics and probiotics (or a synergistic combination of both). Applying prebiotic on day 12 of egg incubation gives the unique opportunity to stimulate endogenous microbiota before hatching. At this time, the chorioallantoic membrane is highly vascularized and allows the transfer of the prebiotic from the air cell into the circulatory system and further to the developing intestine. On the other hand, probiotics enter the GIT the first moment the chick breaks the inner membrane at the early stages of hatching. Therefore, they might act as pioneer colonizers, which augment the development of complex microbiota by modifying the intestinal environment [9]. The same authors claim that pioneer colonizers determine the composition of the climax microbiota by creating the microenvironment necessary for the development of complex microbiota.

**Fig. 1:**
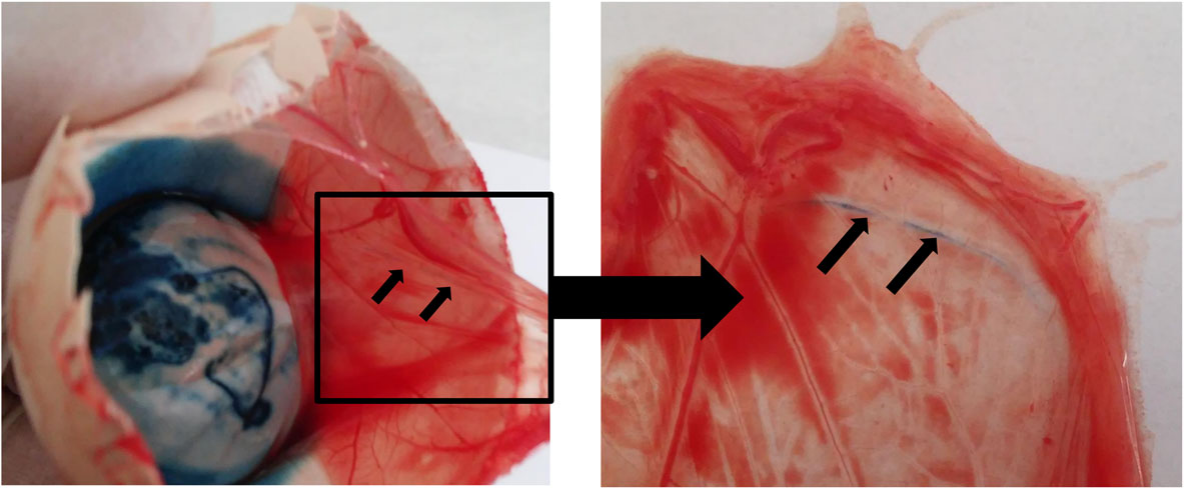
Model of *in ovo* delivery and penetration of the bioactive solution through the chorioallantoic membrane into the circulatory system of the chicken embryo. For *in ovo* injection, 0.2 ml of a blue dye (E132, indigotine, artificial dye used for food coloring) at a concentration of 0.01 g/ml was injected *in ovo* on day 12 of egg incubation. Blue dye was deposited into the air cell. The eggs were sealed and incubation was continued until 19 of egg incubation. Penetration of the dye through chorioallantoic membrane was observed daily by dissecting permeable outer shell membrane from semi-permeable inner cell membrane. After 3 days (i.e., from 15 of egg incubation onwards) the dye was transferred to the blood vessel on the inner shell membrane. The stained vessel is marked with an arrow Steady influx of the dye through the chorioallantoic membrane was observed until 19 of egg incubation, when the experiment was terminated

To demonstrate the transfer of the prebiotic and probiotic after *in ovo* stimulation on day 12 of egg incubation, we performed a simple experiment [34]. The hatching eggs were subjected to *in ovo* delivery of (1) a solution of blue dye with a molecular weight similar to a GOS prebiotic and (2) 10^5^ CFU of a *Lactococcus lactis* subsp. *cremoris* probiotic strain. Both substances were injected into the air cell on day 12 of egg incubation. This approach allowed us to monitor the migration of prebiotic (mimicked by the blue dye) and probiotic through the outer and inner shell membranes. To analyze the migration rate of the bioactives, the eggs were dissected and analyzed daily from day 13 to 18 of egg incubation. We could determine the presence of the blue dye in the embryonic blood vessels by simple observation (Fig. 1). The presence of the probiotic inside the embryo was determined with the plating method. We have demonstrated that that prebiotic/blue dye migrates through the shell membrane and enters the blood circulation on day 3 after injection (i.e., day 15 of egg incubation onwards). Unlike the prebiotic (blue dye), the probiotic bacteria stay in the air cell until the beginning of hatching (i.e., day 19 of egg incubation). Based on the above, we have demonstrated the way and the time point for both prebiotics and probiotics to be delivered *in ovo*. We argue for day 12 of egg incubation as the most optimal time point for the efficient delivery of prebiotic, or prebiotic combined with probiotic (i.e., synbiotic).

#### *In ovo* stimulation—A tool for early programming?

**Fig. 2:**
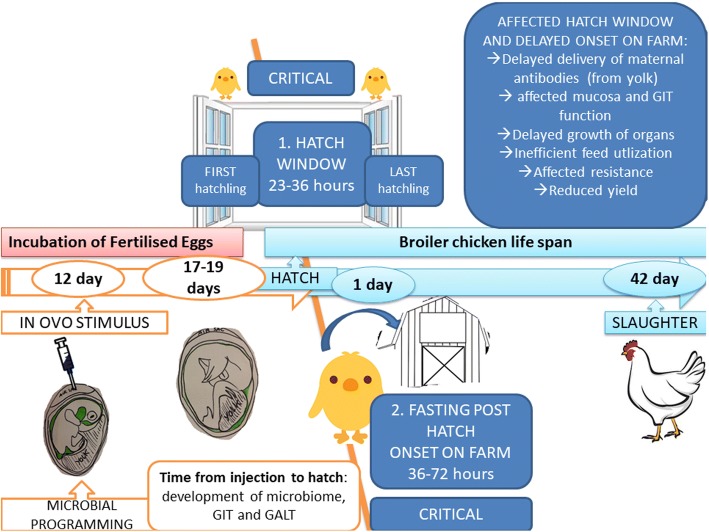
Concept of early microbial programming *in ovo*. Prebiotic or probiotic given on day 12 of egg incubation influences embryonic factors (microbiome, GALT development and function, gene expression, nutrient absorption) which are critical for future phenotype of the broiler chicken. Two critical perinatal moments are shown (hatch window and fasting post-hatching), when the newly hatched chicken is the most receptive to environmental stressors

The concept of perinatal programming was first defined in human studies [35], providing the hypothesis that a stimulus (or adverse factor) provided to a fetus at the early stage of development leads to lifelong phenotypic changes. The animal organism is susceptible to programming at specific developmental stages when it is most receptive to the environment [35]. In poultry practice, two critical moments determine the life-long performance and health status of a bird and these are tightly related with microbiome status: (1) hatching window (the time from when the first chick hatches in the incubator until the last one, usually 48 h) and (2) fasting *post* hatching due to management in a hatchery, transportation [36] and medication treatments [37]. *In ovo* technology seems the only practical tool to provide direct stimuli to a developing embryo to help mitigate the stressors received around the hatch (perinatally) and later in life. Figure 2 presents the critical time points of chicken perinatal development and the life-long production losses that follow. The specific reproduction system of birds allows stimulation of the eggs during incubation in industrial settings. The delivery of prebiotic and probiotic *in ovo* on day 12 of egg incubation stimulates the development of GIT and GALT inside the embryo in response to microbial programming. A newly hatched chick that had been reinforced with *in ovo* stimulation can better handle the perinatal stresses resulting from the hatching window and post-hatching fasting.

#### *In ovo* stimulation vs. *in ovo* feeding

There are different approaches to using *in ovo* technology in poultry, i.e., *in ovo* stimulation (characterized in this paper) and *in ovo* feeding (developed by Uni and Ferket [13]). The differences between *in ovo* stimulation and *in ovo* feeding are fundamental when it comes to the strategy, biological mechanisms and technical tools. *In ovo* stimulation refers to the delivery of prebiotics or synbiotics in the early-stage embryo (day 12 of egg incubation). It aims to stimulate selective growth of indigenous microflora, which colonize embryonic guts. A causative agent that triggers this stimulation is a prebiotic, which is metabolized by the indigenous bacteria, stimulating their growth. Prebiotics and synbiotics can be delivered *in ovo* on day 12 of egg incubation to program the microbiota colonization and modulate gene expression related to the microbiome. Such modulation poses life-long beneficial effects in performance and mitigating life stressors. In turn, *in ovo* feeding is more of a nutritional strategy developed by aiming to enhance chicks’ adaptation to different nutrients post-hatching. In particular, this technology was designed [13] for a late-stage poultry embryo (day 17/18 of egg incubation) to facilitate its adaptation to a switch from embryonic nutrition based on fat and proteins deposited in the yolk (during the embryonic stage) to autonomous nutrition, which is based on carbohydrates and proteins (during the post-hatching stage) [8]. The key parameters, which were measured to evaluate the efficacy of *in ovo* feeding (i.e., the transition from embryonic to independent nutrition), included the proliferation of enterocytes, the enzymatic activity of digestive organs and gut morphology [8]. In summary, *in ovo* stimulation implements a different strategy and underlying biological mechanisms in improving the perinatal development of a chick.

Embryonic development in a chicken is rapid, and the embryo differs from day to day. For this reason, there are differences between *in ovo* stimulation and *in ovo* feeding in the delivery of bioactive compounds. *In ovo* stimulation on day 12 of egg incubation allows for the deposition of the prebiotic/synbiotic solution onto the air cell and the consecutive transfer of small-weight oligosaccharides through the membrane into the blood vessels surrounding the embryo. The prebiotic is transferred through the blood system to the embryo. For this purpose, the prebiotic has to be soluble in water or it does not pass through the membrane. The volume injected is only 0.2 ml, because a larger volume would infiltrate the junction between the inner and outer egg membrane and immediately kill the embryo. *In ovo* feeding of late-term chicken embryos (day 17/18 of egg incubation) must be done directly into the amnion, because at this stage, the air cell is completely dry and devoid of vascularization. The injected volume is larger and amounts to 1–1.7 ml solution of specific nutrients (carbohydrates, proteins and others). The nutrients are swallowed by the embryo with the amniotic fluid and are made available through the intestinal tract. When we consider the distinction in the route of administration between *in ovo* stimulation and *in ovo* feeding, we need to acknowledge that only prebiotic compound is used for actual stimulation of the indigenous flora in the embryo. Probiotic, even though it might be injected on day 12 of egg incubation (in the form of synbiotic), should be considered *in ovo* feeding due to its availability to the late-stage embryo (after pipping).

Manipulation of the eggs at different stages of incubation requires the development of different technological strategies. In industrial settings, the most common and sensible time point for performing *in ovo* injection is day 18 of egg incubation. At this time, the eggs are candled and transferred from the incubator to the hatcher. The most common application of *in ovo* technology on day 18 of egg incubation is *in ovo* vaccination against Marek’s disease virus. The attempts to combine *in ovo* vaccination with *in ovo* feeding has been scarce and it is hard to predict its effectiveness [38]. The injection machines that are available on the market are designed to perform intramuscular injection with the vaccine (e.g., www.egginject.com) on days referred to as the “*in ovo* injection window” (i.e., days 17–19 of egg incubation). These machines generally do not support amniotic delivery of nutrients (or other bioactives) due to the high probability of an imprecise injection site by the operating needle. The possible imprecision in targeting the amnion of late-term embryos is due to several factors, such as (1) the design of the needles in vaccination systems, (2) imperfectly controlled positioning of injected eggs, and (3) the fact that each incubated embryo uses and/or rearranges the compartments of its egg (air cell, allantoic sac, amniotic sac, yolk sac) in a very fast and individual manner in the later stage of incubation. The embryo is already filling almost the whole space of the egg on days 18–19.5 of egg incubation, which makes it harder to locate the amnion for automated *in ovo* feeding. Using early-stage embryos for *in ovo* injection is much more standardized and safer for the viability of the embryos. On day 12 of egg incubation, the air cell is large and easily available for the injection without having the embryo injected by a needle. For this reason, *in ovo* stimulation does not dampen hatchability results. *In ovo* stimulation provides beneficial and long-lasting effects to the embryo but it is still a new technology on the market. The major obstacle to using *in ovo* stimulation is the need for technological adjustment of production lines in hatcheries to add another time point for egg manipulation (day 12 of egg incubation). The injection machine for *in ovo* injection of early-stage embryos needs to be adjusted so that the injection is done into the air cell and the puncture hole is sealed to avoid embryo evaporation. A prototype of such an automated injection system for early-stage embryos exists and has been tested in commercial settings [39]. The pipeline includes three procedures: a hole is drilled in the eggshell, a solution is injected, and then the hole is sealed. This allows for *in ovo* injections of 30,000 eggs per hour while maintaining superior hatchability.

In summary, there are pros and cons to using *in ovo* stimulation with prebiotics or *in ovo* feeding with probiotics for embryonic microflora development in chicken. Both approaches require adjustment in the production line and additional handling of the eggs. However, *in ovo* stimulation is done on day 12 of egg incubation, and therefore it cannot be combined with other procedures (e.g., vaccination). On the other hand, *in ovo* stimulation assures stable and long-term effects compared with the more transient effects of *in ovo* feeding. Also, through *in ovo* stimulation we enhance the growth of indigenous, diverse microbial populations, which is biologically more relevant than delivering a single (or even multistrain) inoculation with arbitrarily selected microbiota. This is how we claim that the long-term effects on *in ovo* stimulation are based on natural mechanisms of early gut colonization and can provide a valuable addition to the perinatal treatment of chicks.

### Life-long phenotypic effects

Life-long phenotypic effects that followed *in ovo* stimulation were determined on multiple levels, from industrially relevant traits, such as hatchability, mortality and performance among different genotypes of broiler chickens (e.g., Ross, Cobb and Hubbard) and native chickens (i.e., Green-legged Partridgelike), through the morphology and histology of the intestinal, muscular and immune tissue, to physiological and molecular modulation. In this chapter, we discuss the life-long phenotypic effects of microbiome stimulation with prebiotics or synbiotics administered *in ovo* on day 12 of egg incubation. The results presented here were derived from a series of experimental and industrial trials and give quite a good outlook on the effects of *in ovo* stimulation in chickens (Table 1). However, many trials are still in progress to complement the picture with data on the metagenome and challenge response of chickens stimulated *in ovo*.

**Table 1:** Overview of the life-long phenotypic effects of *in ovo* stimulation with prebiotics and probiotics on day12 of egg incubation

Prebiotic	Amount (mg/ml)	Probiotic	Amount CFU	Effects of *in ovo* stimulation	Reference
a. Hatchability, performance and intestinal microbiota					
RFO	0,1763; 0,8815; 1763	–	–	Confirmed effects of RFO injected *in ovo* on day 12 of egg incubation, increased number of bifidobacteria	Villaluenga et al. 2004 [11]
FOS, RFO	1763 2,1158	–	–	Decreased mortality, high level of bifidobacteria after hatching and before slaughter	Pilarski et al. 2005 [39]
RFO	1900	–	–	Increased body weight and FCR, increased and dose-dependent counts of bifidobacteria	Bednarczyk et al. 2011 [38]
RFO	1900	–	–	Improved performance traits of broiler chickens	Brudnicki et al., 2015 [40]
GOS, *Laminaria* spp. seaweed extract containing laminarin and fucoidan	3500 0,880	–	–	Increased number of lactobacilli and bifidobacteria in chicken feces, validation of *in ovo* vs. in-feed prebiotics administration	Bednarczyk et al., 2016 [33]
GOS, *Laminaria* spp. seaweed extract containing laminarin and fucoidan	3500 0,880	–	–	Improvement in a number of production parameters including carcass weight and yield	Maiorano et al., 2017 [41]
GOS, RFO	3500 1900	*Lactobacillus salivarius, Lactobacillus plantarum*		Modulation of the fecal microflora composition	Dunislawska et al.,2017 [42]
b. Intestinal morphology					
RFO	1500 3000 4500	–	–	Potential to enhance ileum mucosa morphology and improve immunity in the small intestine	Berrocoso et al., 2016 [43]
Inulin, GOS	1760 0,528	*Lactococcus lactis* subsp. *lactis, Lactococcus lactis* subsp. *cremoris*	10^3^ 10^3^	Increase in the number of goblet cells in the duodenum and the jejunum on day 1 of life, followed by significant decrease on the day 4	Bogucka et al., 2016 [44]
Inulin, GOS	1760 0,528	*Lactococcus lactis* subsp. *lactis, Lactococcus lactis* subsp. *cremoris*	10^3^ 10^3^	Significantly higher number of goblet cells	Bogucka et al., 2017 [45]
RFO	1900	–	–	Increased length of the small intestine and transient effect (days 1–3 post-hatching) on villi length and surface	Brudnicki et al., 2017 [46]
*Laminaria* spp. seaweed extract containing laminarin and fucoidan	0,880	–	–	Increase in villi width and crypt depth in duodenum on day 21	Sobolewska et al., 2017 [47]
GOS, RFO	2000 2000	*Lactobacillus salivarius, Lactobacillus plantarum*	10^5^ 10^5^	Increased absorbent area of villi in different sections of the intestine on days 1 and 42, increase in the number of the goblet cells, reduced crypt depth	Sobolewska et al., 2017 [48]
c. Muscle histology and meat quality					
RFO	1900	–	–	Improved meat quality in terms of collagen content	Maiorano et al., 2012 [49]
d. Immune system development and function					
Oligosaccharide extract of palm kernel cake (PKC)	20	–	–	Increased IgG production and antioxidant capacity in serum and liver of prenatal chicks and had limited carrying-over effects on the post-hatched chicks	Jahromi et al., 2017 [50]
Inulin, GOS	1760 0,528	*Lactococcus lactis* subsp. *lactis, Lactococcus lactis* subsp. *cremoris*	10^3^ 10^3^	Prebiotics and synbiotics can modulate the central and peripheral lymphatic organ development in broilers	Madej et al., 2015 [51]
Inulin, GOS	1760 0,528	*Lactococcus lactis* subsp. *lactis, Lactococcus lactis* subsp. *cremoris*	10^3^ 10^3^	Stimulated GALT development after hatching and colonization with lymphocytes	Madej & Bednarczyk 2016 [52]
RFO	0,7765 0,8815 1763	–	–	Protective effect against *Salmonella enteritidis* infection	Ruiz Lopez et al., 2008 [53]
e. Gene expression modulation					
RFO	1900	*Lactococcus lactis* subsp. *lactis, Lactococcus lactis* subsp. *cremoris,*	10^3^ 10^3^	Stimulation of an immune system by lymphatic organs development	Slawinska et al., 2014 [54]
Inulin	1760	*Lactococcus lactis* subsp. *lactis,*	10^3^	Down-regulation of gene expression in cecal tonsils and spleen	Plowiec et al., 2015 [55]
Inulin GOS	1760 0,528	*Lactococcus lactis* subsp. *lactis, Lactococcus lactis* subsp. *cremoris*	10^3^ 10^3^	Long-term transcriptomic effects	Slawinska et al., 2016 [56]
GOS, RFO	2000 2000	*Lactobacillus salivarius, Lactobacillus plantarum*	10^5^ 10^5^	Significant impact on gene expression level in spleen and cecal tonsils	Dunislawska et al., 2017 [42]
GOS, RFO	2000 2000	*Lactobacillus salivarius, Lactobacillus plantarum*	10^5^ 10^5^	Downregulated GLP-1 and GIP mRNA expression in the duodenum and GLP-1R in the pancreas	Kołodziejski et al., 2018 [57]
f. Avian physiology					
RFO	1900	–	–	Increased rate of yolk sac absorption	Brudnicki et al., 2015 [40]
Inulin GOS	1760 0,528	*Lactococcus lactis* subsp. *lactis, Lactococcus lactis* subsp. *cremoris*	10^3^ 10^3^	Increased the activity of amylase, lipase, and trypsin in the pancreas	Pruszynska Oszmalek et al., 2015 [58]
Inulin GOS	1760 0,528	*Lactococcus lactis* subsp. *lactis, Lactococcus lactis* subsp. *cremoris*	10^3^ 10^3^	Increased body weight, improved the short – chain fatty acid cecal profile, increase the villus length	Miśta et al., 2017 [59]

#### Hatchability, performance and intestinal microbiota

One of the major concerns related to *in ovo* technology is its impact on egg hatchability and chick mortality. Hatchability depends on the incubation conditions, *in ovo* delivery technique, site and depth of injection (air cell vs. amnion), and the kind of bioactive and inoculated dose [8, 39, 40]. In some cases, especially when late-term embryos are handled or the dosage of the bioactive is exceeded, hatchability may drop rapidly, which decreases hatching success [40]. In general, properly optimized doses for *in ovo* stimulation will keep hatchability at a high level and improve bifidobacteria counts [11, 33]. In relation to the performance traits of broiler chickens, including body weight, feed intake and efficiency, carcass traits and meat quality, the results, though different between trials, showed very slight changes in *in ovo*-stimulated chickens in comparison to controls. But, our validation study conducted in industrial settings (as opposed to small-scale experimental trials that are usually reported) on 275,000 broilers delivered a proof of concept that *in ovo* stimulation with prebiotics in fact improves performance traits, including body weight (BW), carcass weight, carcass yield, and breast muscle weight [41]. But, the aforementioned data were collected under controlled conditions and the birds were not challenged with any environmental or immunological stresses. In our ongoing challenge study, we have detected a large improvement in *in ovo*-stimulated birds in mitigating the harmful effects of heat stress, expressed by significantly decreased mortality, faster recovery of feed intake and body weight gain and decreased number of myopathies in the meat [42]. We are convinced that further exploring the effects of *in ovo* stimulation on challenged chickens will prove them more resilient to handle stressful environments in a more robust way.

#### Intestinal morphology

The morphology of the intestinal mucosa is an important determinant of the digestive and absorptive intestinal functions, which in turn determine the growth performance of poultry. In our research, *in ovo* stimulation with prebiotics in combination with probiotics affected the histomorphology of the small intestine of chickens. The observed effects strictly depend on the type of bioactive substances used for *in ovo* stimulation and the age of the chicken. The effects of *Lactobacillus*-based synbiotics on histomorphology were assessed on days 1 and 42 post-hatching. On day 1, both synbiotics, *L. salivarius* (combined with prebiotic GOS - SYN1) and *L. plantarum* (with RFO - SYN2) increased villus height, width and surface in the duodenum of Cobb broiler chickens. Increasing the absorbent surface by increasing the surface of the intestinal villi as a result of SYN1 injection was also observed in the duodenum of chickens at 42 days of age. A similar effect of the synbiotic used was found in the jejunum in 1-day-old chicks. On day 42 post-hatching (end-point of the study), small intestinal length and weight, as well as histomorphology of the duodenum (increasing the width of villi, surface area and deepening of intestinal crypts), was improved after *in ovo* delivery of *L. salivarius* (with GOS). Additionally, it is worth mentioning that this synbiotic increased the number of neutral goblet cells in the jejunum and ileum. [43]. In intestinal crypts, epithelial cells undergo intensive mitotic divisions. Deeper crypts allow for faster regeneration of intestinal villi. The depth of the crypt is associated with faster turnover of epithelial cells and thus also goblet cells. Goblet cells produce mucus, which constitutes one of the components of the physical barrier in the guts [44, 45]. *Lactococcus*-based synbiotics had more transient effects. Their positive effects were found in the small intestine of Ross broiler chickens on the first days post-hatching but were different depending on the investigated section of the small intestine and age of chickens. On the first day of life, an increase in the height of the villi in the jejunum was found as a result of the injection with prebiotics (inulin and GOS) and synbiotics *L. lactis* spp. *lactis* with inulin and *L. lactis* spp. *cremoris* with GOS. The stimulatory effect of both synbiotics on the villus surface area of the duodenum was also reported on days 1 and 4 post-hatching and of the jejunum on day 4 post-hatching. In the majority of the examined traits of the small intestine morphology, a positive effect of *in ovo* injections of synbiotics was demonstrated [46, 47]. At the end of rearing (day 35), a positive effect on the histomorphology of the small intestine was exerted by inulin as it increased the villus height in the duodenum as well as ileum (the main absorption organ). The absorptive area of the duodenum increased in response to inulin supplementation. In the jejunum and ileum, the villus surface area was negatively affected only by the synbiotic with the GOS preparation, but other injected substances had a positive effect on the absorptive area of the intestine. Chickens that received *in ovo* synbiotic with inulin (jejunum) and the synbiotic with GOS had deeper crypts in the jejunum and ileum, respectively. The injection of synbiotics also increased the number of goblet cells (synbiotic with GOS) in the jejunum and ileum [47], which suggests it has regulatory effects on intestinal morphology. In our other studies, the positive effect of GOS and RFO prebiotics on the width and area of the villi of Ross broiler chickens at day 21 post-hatching was found (Bogucka, personal communication). Moreover, it has also been demonstrated that the injection of an extract of *Laminaria* spp. containing laminarin and fucoidan significantly increased the width of the duodenal villi and the crypt depth of chickens on day 21 of rearing. This resulted in a greater surface area of villi in these birds; however, this was not confirmed statistically [43].

#### Muscle histology and meat quality

The effect of bioactive substances administrated *in ovo* on the meat quality and microstructure of birds’ skeletal muscles is not homogeneous. However, an increasing number of studies suggest there is a relation between the gut microbiome, the metabolic pathway of substances absorbed in the intestine, and a later increase in muscle mass and the formation of the quality traits of meat, although the mechanisms of these processes are still difficult to identify. Maiorano et al. [48] studied the influence of prebiotic and three different synbiotics on the chicken pectoral muscle microstructure. They observed a decrease in the density of the muscle fiber per mm^2^ in the synbiotic group (RFO with *L. lactis* spp. *cremoris*) in comparison to the control group. Moreover, intramuscular collagen was notably reduced in the prebiotic and synbiotic groups. There was no effect of synbiotics on abdominal fat, ultimate pH, and cholesterol in the pectoral muscle of chickens. In other studies that aimed to explore the effect of GOS and extract of *Laminaria* spp. injected *in ov*o, there was a considerable reduction in the diameter of the muscle fibers in the prebiotic groups; however, this was not confirmed by statistical analysis. Furthermore, meat from prebiotic-treated birds displayed higher lipid oxidation levels compared to those from the control during the entire storage time [41]. Intramuscular fat content has an effect on the flavor and juiciness of meat, as well as the visual aspect (marbling). Sometimes, the increased percentage of fat in the pectoral muscle of broiler chickens may be associated with the occurrence of meat defects. They are manifested by numerous pathological changes of muscle fibers leading to their necrosis, which may result in fibrosis and increased fat content with a modified fatty acid composition, reduced protein content, which leads to a decrease in the nutritional value of meat [49–51]. In the white meat of broiler chickens, lipids accumulate in the connective tissue surrounding the fibers and bundles of fibers. Dankowiakowska et al. [52] showed the effect of GOS prebiotic on increasing the intramuscular fat content in the breast of Ross 308 chickens. In addition, the *in ovo* injection of synbiotic (GOS with *L. salivarius*) has been shown to have a positive effect on the blood supply of broiler chickens’ pectoral muscle. There were statistically more capillaries per unit area and per one muscle fiber in the synbiotic group. Better muscle capillarity influenced less occurrence of pathological changes, i.e., fiber necrosis and splitting, and hence, a higher percentage of normal fibers (Bogucka, personal communication). A similar effect of synbiotic dietary supplementation (RFO with *Lactococcus lactis*, *Carnobacterium divergens*, *Lactobacillus casei*, *Lactobacillus plantarum*, and *Saccharomyces cerevisiae*), obtained in *pectoralis major* muscle of female broiler chickens [53]. As it turns out, not only the type of bioactive substances but also the way they are administered may affect the microstructure of the muscles and the quality of the meat. Tavaniello et al. [54] studied the effect of prebiotics administration on the meat-quality traits of broiler chicken by evaluating the different routes of their delivery (*in ovo* vs. in-water vs. *in ovo* and in-water combined). Irrespective of the delivery method, prebiotics showed a positive impact on breast muscle weight and yield, which was also associated with a greater thickness of muscle fibers. The redness (a*) of fillets was statistically decreased upon giving prebiotics, irrespective of the method used. In addition, the authors showed a higher content of PUFA (polyunsaturated fatty acids) and n-3 fatty acid in the meat from the prebiotic groups, displaying more favorable indexes for human health. Similar studies were conducted by Tavaniello et al. [55], which compared the effect of *in ovo* administration of two different synbiotic formulations (GOS with *L. salivarius* - SYN1 and RFO with *L. plantarum* - SYN2) on carcass- and meat-quality traits in broiler chickens. The highest color lightness (L*) in 45 min after slaughter is characteristic of meat from *in ovo* SYN1-injected chickens. In contrast, in the SYN2 group, the highest content of MUFA (monounsaturated fatty acids), PUFA (polyunsaturated fatty acids) and n-6 fatty acid was recorded.

#### Immune system development and function

It was demonstrated that *in ovo* stimulation with prebiotics and probiotics delivered on day 12 of egg incubation influences immune system development, including the structure of the central (i.e., bursa of Fabricius and thymus) and peripheral (i.e., spleen) lymphatic organs [56–58]. *In ovo* application of synbiotics provide stimulus for the immune organs of the growing chickens, but the potency of the stimulation depends on the chicken genotype. The bursa and bursa-to-spleen index were significantly higher in broiler chickens after *in ovo* stimulation with RFO and *Lactococcus lactis* subsp. *cremoris*. In Green-legged Partidgelike chicken, the spleen index was higher, but only on day 21 post-hatching. The histological image of the thymus displayed the increased density of thymocytes in the cortex after *in ovo* stimulation with two different synbiotics [57]. More robust immune system development is beneficial for mounting immune responses later in life. At hatching, the GALT is immature and requires early stimulation, otherwise it will impair the health status and performance of the animal [59]. To address this issue, a bioactive stimulus provided on day 12 of egg incubation may influence, through modulation of gene expression [60], developmental events related to future immunological functions. The more so because the precursors of T and B lymphocytes, major players in cellular (T) and humoral (B) immune response, are established between the 9th and 15th days of egg incubation and begin to differentiate and proliferate continuously in immune organs until several days post-hatching [61].

*In ovo* stimulation with synbiotics influences the immune phenotype and cell distribution in the cecal tonsils, ileum and bursa of Fabricius of broiler chicken [58]. These tissues were immunohistochemically stained for the estimation of Bu-1+, CD3+, CD4+, CD8α + and TCRγδ+ properties. The results indicated that synbiotics stimulated post-hatching development of GALT in chickens. A temporary decrease in the B-cell number in the bursa of Fabricius on day 7 post-hatching was detected, which can suggest an increased colonization rate of the peripheral lymphoid organs as an effect of *in ovo* stimulation with *Lactococcus lactis* subsp. *cremoris* and GOS [58]. The chicken immune system undergoes rapid changes during its first weeks post-hatching; some effects, such as an increase in the spleen/bursa Fabricius ratio, were age-dependent [56]. In the cecal tonsils, a high colonization of GALT by T cells was observed after *in ovo* stimulation with two different synbiotics: *L. lactis* subsp. *lactis* combined with inulin and *L. lactis* subsp. *cremoris* with GOS [57]. These synbiotics stimulated also germinal center formation in the spleen, observed on days 21 and 35 post-hatching, which indicated enhanced B-cell proliferation in peripheral lymphatic organs.

#### Gene expression modulation

The injection of synbiotics into the air cell on day 12 of egg incubation effectively stimulates the host microbiome, which indirectly influences changes in gene expression in broiler chickens. A single bioactive dose applied *in ovo* allows for early intestinal contact with bacteria by the direct stimulation of the host microbiome and an indirect effect on the modulation of the host transcriptome. Probiotic bacteria *Lactococcus lactis* combined with RFO prebiotic had a significant effect on the gene expression of cytokines and chemokines in cecal tonsils (gene expression down-regulation) and spleen (gene expression up-regulation) of Green-legged Partridgelike chickens [62]. Further experiments on *Lactococcus lactis* combined with inulin prebiotic, performed in a time-course manner, proved that *in ovo* stimulation down-regulated immune-related gene expression in cecal tonsils and spleen of broiler chickens and those effects were more pronounced in older animals (day 42) [63]. Analysis of the spleen, cecal tonsils and large intestine transcriptome of chickens that received *Lactococcus lactis* combined with prebiotic (GOS or inulin) allowed a determination of the transcriptome modulation profile after the synbiotics administration [60]. The highest number of differentially expressed genes was detected after *in ovo* stimulation with GOS prebiotic. The activated molecular pathways took part in different immune processes, such as the Toll-like receptor signaling pathways, TCR signaling, BCR signaling, NF-KB signaling, hematopoietic cell lineage, TNF signaling, G-coupled protein signaling and cytokine signaling [60]. *Lactobacillus salivarius* combined with GOS and *Lactobacillus plantarum* combined with RFO showed involvement in incretin secretion and reception defined by analysis of the mRNA level for GLP-1 and GIP [64].

#### Avian physiology

Supplementation of probiotics aims to replace or reduce the number of potentially pathogenic bacteria in the intestine by enriching the populations of beneficial strains. It improves the health status of the intestine and may be reflected in the general metabolism, as well as organ-specific biochemical processes.

Current research shows that the functioning of the liver, fatty tissue, kidney, and pancreas can be affected by disorders of the intestinal microflora. The increase in enzyme activity may be related to the delivery of additional portions of the enzyme by bacteria living in the intestine, which causes an improvement in nutrient digestibility and an increase in body weight [65]. Pruszynska-Oszmalek et al. [66] demonstrated that the beneficial effects of *in ovo* stimulation with *Lactococcus lactis* probiotics combined with inulin or GOS prebiotic significantly increased the total activity of pancreatic enzymes—amylase, lipase, and trypsin—and, as a consequence, increased the chickens’ body weight. Moreover, these authors show a positive effect of both synbiotics on the activities of the two enzymatic markers of the liver (aminotransferase: ALT and AST), which demonstrates the high health status of the liver.

Brudnicki et al. [67] demonstrated that *in ovo* stimulation of broiler chickens with RFO prebiotic significantly increased the yolk sac resorption rate in newly hatched chicks. By the end of day 14 post-hatching, the retention of the yolk sac in the population was 0% in the *in ovo*-stimulated group vs. 30% in the control group. Since the yolk sac is the major source of immunoglobulins contributing to the passive immunity in newly hatched chicks, faster yolk sac resorption results in the greater transfer of maternal antibodies into the chicks’ blood stream. Also, the yolk sac is essential for the initiation of early growth post-hatching in broiler chickens [68]. In the aforementioned study, body weight and feed efficiency were indeed improved at the end of the study (day 42). Finally, we have demonstrated that *in ovo* stimulation improved the profile of short-chain fatty acids in the ceca of broiler chickens, expressed by a higher molar percentage of butyrate, propionate and valerate, especially in older birds (21 and 35 days post-hatching).

#### Future perspectives

In this paper, we gave insight on microbiome programming with the early delivery of prebiotics and probiotics during the neonatal period in chickens. We have demonstrated that *in ovo* stimulation is a powerful tool for the early colonization of the embryonic guts with beneficial microbes so that it results in improved productivity, health and welfare of the animals. There are still questions ahead that will drive the development of prebiotics and probiotics for animals even further. Being aware of the miscellaneous effects of different probiotic formulations, it is tempting to fit the bacteria strain to the specific needs of the individual animal, depending on environmental pressure, host genetics and production goals. Currently, a screening procedure for prebiotics and probiotics is carried out with different in vitro, in vivo and “-omics” approaches and includes response to stresses within the host, ability to adhere to the intestinal wall and colonize the guts, and beneficial functions such as antimicrobials production, metabolism stimulation and immunomodulation. In an excellent review paper, Papadimitriou et al. [69] presented a number of currently available methodologies applied to select safe and effective probiotic strains.

But even now that worldwide research is focused on handling microbiome composition and function, there is a knowledge gap in the precise prediction of new probiotics from known collections using conventional approaches [70]. These are also limited by the oxygen conditions used to co-culture aerobic strains with eukaryotic host cells in vitro, and as such omit the majority of gut microbiota, which are anaerobic. Technological development in this field resulted in modeling artificial guts for the simulation of the complete digestive process, from ingestion of bacteria, passing through different compartments of the GIT, to fermentation in the hindgut. With this, complex microbiome studies including interactions with the host can be carried out also in anaerobic conditions, which was reviewed elsewhere [71, 72]. One of the advanced gut models, referred to as TIM-2 (TNO computer-controlled, dynamic in vitro gastro-Intestinal Model of the colon), which was developed for human research [73] is now being adjusted to mimic chicken guts and will be used in poultry research [74]. Another line of technological advancements in microbiome research has been facilitated by nanotechnology and led to the development of gut-on-a-chip [75]. This microfluidic device allows for growing human Caco-2 intestinal epithelial cells under constant fluid flow and peristatic movements mimicked by vacuum chambers. Intestinal epithelia grown on gut-on-a-chip formed faster and were much taller than in other in vitro assays, the morphology typically found only in human epithelia in vivo.

In case advanced selection methods of potential probiotics do not suffice, bacteria can be engineered to contain a specific trait. First, probiotics were biotechnologically “designed” to express phenotype of interest, such as more efficient fermentation of prebiotic fibers, producing lactic acid, resistance to stress or improved adherence to intestinal mucosa. Some of those probiotics were modified to mimic a pathogen’s survival strategy and they were all called “bioengineered bugs” [76]. More recently, the emergence of synthetic biology revolutionized this field, bringing newly designed and synthesized biological systems. This technology allows engineering microbes with novel therapeutic functions, such as a “sense-and-kill” strategy towards specific infectious agents or cancer cells [77]. Even though this approach is very sophisticated and currently used only in biomedical studies, antibiotic crisis in livestock followed by outbursts of infectious diseases and the need for new therapeutic approaches, may result in applying designer probiotics in animals.

Biotechnology is also used to produce next-generation non-digestible oligosaccharide prebiotics (NGOs), which was reviewed by Mano et al. [78]. A vast majority of oligosaccharide prebiotics is produced by hydrolysis of the naturally occurring polysaccharides. The specific enzymes that transform polysaccharides into NGOs are derived from microbes, which very often is insufficient. For this reason, oligosaccharide biotechnology is based on the development of the optimal bacterial or enzymatic system to process sugars into NGOs. Only properly balanced oligosaccharide prebiotics will pass the small intestine and undergo microbial fermentation in the hindgut. They can be extremely immunoactive and can be used to target literally any microbial community. To meet the increasing demand for prebiotics, new materials are being explored as sources for obtaining NGOs. They include biomass of industrial byproducts of different plants, such as apples, soy, carrot, beans and other malting produce (reviewed by Patel et al., 2012 [79]). Also, new super-plants from different cultures are being investigated, such as yacon, blueberry, pleuran mushrooms and green tea, just to mention a few. It appears that in nature there are many more novel sources of bioactive NGOs. Last but not least are resistant starches, even though they are not prebiotics per se but have become recognized as potent fermentation substrates of many microbes. Resistant starches are able to escape undigested from the small intestine only to be fermented in the large intestine and become a rich source of SCFA (especially butyrate) production [80]. In this way, resistant starches, which are inexpensive and ubiquitous, might be considered prebiotics of the future.

An alternative approach that would not require careful selection of the most beneficial microbes is to transplant the whole microbiome from individuals of preferred phenotype to recipient ones. This method, called fecal microbiota transplant (FMT), was recently tested on chicken lines that differed in feed efficiency [81]. The newly hatched chicks from the line of poor feed efficiency received FMT from a highly efficient line. In this study, FMT allowed colonization with specific feed efficiency associated bacteria, but did not affect the chicken phenotype as much. These data, though promising, have proven that microbiome transplantation is still an immature technique. Also, future issues associated with FMT include better control over microbial composition and developing a method (e.g., encapsulation) to preserve the transplants before use.

By definition, probiotics contain viable microorganisms because their efficiency is strongly correlated with the ability to colonize the guts. For this reason, stabilizing the probiotics so they can retain their viability after production processes and storage is a must. The core technology is to immobilize the live bacteria using an inert material. The protection strategy depends on the properties of the probiotic strain, such as the range of tolerance to pH, temperature and oxygen, and the stresses that may affect its viability in the intestine [82]. One of the emerging technologies for the immobilization of probiotics for livestock is probiotic encapsulation technology (PET), which uses a range of materials, from a common carbohydrate matrix to hydrogel-based delivery systems [83]. If the effort into selecting (or engineering) the proper probiotic is becoming more advanced and sophisticated, the PET should follow to protect the microbes during production, storage and ingestion. And the need for more advanced PET technologies will introduce a significant upgrade into feed mills.

When discussing the future of prebiotics and probiotics in animals, one cannot forget about postbiotics, a term that refers to the byproducts of bacterial metabolism, which have biological activity in the host. Postbiotics include bacteriocins, exopolysaccharides, vitamins, and short-chain fatty acids. They exert beneficial effects on the host without the risk of delivering live bacteria (e.g., horizontal transfer of antibiotic resistance genes) [84]. Killed probiotics are sometimes considered postbiotics, because they are still able to modulate the immune system. In a practical sense, postbiotics are much easier to standardize and deliver in encapsulated form. They also fulfill all safety standards, but the effects of using postbiotics may be transient compared to probiotics. Therefore, some authors report using postbiotics in combination with prebiotics to include long-term beneficial effects in the stimulation of indigenous flora [85].

Last but not least, when considering probiotics in animal production as a dietary supplement with health benefits, the cost of such supplementation is an important factor to consider. Therefore, there is an interest in a transgenerational effect of probiotics applied in livestock. Berghof et al. [86] published a very interesting review paper on transgenerational epigenetics effects on innate immunity in broiler chickens. The authors propose two possible scenarios for modulation of transgenerational effects in broiler chickens, including direct modulation of the chicken embryo or passing on transgenerational effects from the stimulated dams to their offspring. Both of those cases are interesting from an *in ovo* stimulation point of view. First, as we tried to document in this paper, early stimulation of the native microflora in chickens modifies the embryo environment, especially microbial population, which has an intrinsic role in embryo stimulation. Research has not been conducted so far to determine the molecular epigenetic effects at the DNA level. However, we have determined numerous effects of *in ovo* stimulation on different characteristics of the bird, including its transcriptome modulation. It is still unclear if we can successfully stimulate dams *in ovo* so that offspring can generate beneficial effects. It is quite crucial to emphasize that the transgenerational effects of *in ovo* stimulation can express themselves as the phenotype, for example, an increased level of maternal antibodies in the egg yolk or beneficial microbes stored at the surface of the eggshell. Hopefully, future research will shed some light on those questions.

## Conclusions

In this review paper, we tried to synthesize the concepts and advancements of *in ovo* stimulation of chicken microflora by delivering prebiotics and probiotics into the air cell on day 12 of egg incubation. In a chicken embryo model at this stage, a bioactive substance deposited inside the air cell can enter the embryonic GIT from two different routes. Oligosaccharide prebiotics that are soluble in water can pass the outer and inner shell membranes and reach the embryo through the blood stream. This process takes place between day 12 and 18 of egg incubation following a single-dose injection with prebiotic. On the other hand, probiotic is engulfed by the embryo on day 19 of egg incubation, during the early phases of hatching. Carefully selected bioactives, delivered *in ovo* on day 12 of egg incubation is the earliest method to stimulate intestinal environment and trigger GALT maturation in chickens. Since it is delivered early in embryonic development, it exerts a strong modulatory effect on the embryo. The determined, so far life-long, phenotypic modulation includes performance traits, intestinal development and microflora abundance at hatching, immune system development, increased SCFA production, yolk-sac absorption and gene expression signatures regulation. The effects were stable and expressed up to six weeks post-hatching, which is the endpoint of the broiler trials. We are convinced that this method can be introduced into commercial settings, given the possible introduction of dedicated infrastructure compliant with production lines used in hatcheries. The ongoing studies in this area consider mitigating effects in chickens under environmental and immunological challenges as well as transgenerational effects that could increase the impact of *in ovo* stimulation.

## Abbreviations

CFU: Colony-forming units
GALT: Gut-associated lymphoid tissue
GIT: Gastrointestinal tract
OUT: Operational taxonomic unit
SCFA: Short-chain fatty acids
